# Comparison of Snap Traps and Cages for Trapping *Rattus norvegicus* in Urban Residential Buildings

**DOI:** 10.3390/ani16121805

**Published:** 2026-06-11

**Authors:** Babatunji Daramola, Changlu Wang

**Affiliations:** Department of Entomology, Rutgers University, 96 Lipman Drive, New Brunswick, NJ 08901, USA; bsd70@scarletmail.rutgers.edu

**Keywords:** *Rattus norvegicus*, trapping efficacy, demographic bias, rat surveillance, rat management

## Abstract

Mechanical traps are widely used for urban rat surveillance and management, including the brown rat, *Rattus norvegicus* (Berkenhout). However, relatively few studies have examined the effects of environment, placement, and trap type on trapping outcomes in residential buildings. In this study, we deployed 50 pairs of snap traps and live cage traps for five nights (500 trap nights) in concrete-covered basements in Brooklyn and dirt-covered crawlspaces in Queens, New York City, USA. Capture success differed by site, with Brooklyn yielding higher overall success (38%) than Queens (13%) (*p* < 0.001). Capture success in Brooklyn was lower in compactor rooms than in other areas (vertical infrastructure, open-floor travel corridors, and burrow entrances). In Queens, success was similar at different placement areas. The two trap types had similar overall capture success (*p* = 0.090). Cage traps produced more female-biased samples (90% females), while snap traps yielded more balanced sex ratios (53% females) (*p* < 0.001). In addition, median body mass distributions also differed, with snap traps capturing heavier rats than cage traps at both sites. These findings demonstrate that both placement and trap design are important considerations for urban rat surveillance and urban rat management.

## Simple Summary

Brown rats are a major pest in urban environments, causing structural damage, contaminating food and spreading diseases. While snap traps and cage traps are widely used to catch them for disease surveillance and control, it is not well understood how different indoor environments and trap designs affect trapping success in urban residential buildings. In this study, we compared the performance of snap traps and live cage traps in concrete basements and dirt crawlspaces in residential buildings in New York City. A total of 24 pairs of traps were placed in basements, and 26 pairs of traps were placed in crawlspaces. Across 500 trap nights, capture success was 38% in basements and 13% in crawlspaces. Although both trap types had similar overall success rates, cage traps primarily caught younger, female rats, whereas snap traps caught a more balanced ratio of males and females, as well as heavier, older rats. These findings provide valuable insight for public health and pest management professionals, helping them select the most effective trapping strategies to monitor and control urban rat populations.

## 1. Introduction

Brown rats, *Rattus norvegicus* (Berkenhout), are one of the most economically and epidemiologically significant vertebrate pests in metropolitan environments worldwide [1]. Their persistence in densely populated urban settings contributes to structural damage, food contamination [2,3], and the maintenance and transmission of zoonotic [4,5,6] and ectoparasite-borne pathogens [7]. As a result, effective surveillance and control of the rat populations remain central objectives of applied pest management and urban ecology [6,8,9].

Mechanical trapping remains a key part of brown rat surveillance [10] and control programs [1] with placement strategies often prioritizing floor-wall junctions and active runways [11]. Among available mechanical tools, lethal snap traps and live-capture cage traps are some of the most used [1,10,12]. Snap traps are commonly favored in operational pest management due to their low cost, rapid lethality, relatively low animal welfare impact, and ease of deployment [11,13], whereas cage traps are essential when live animals are required for biological sampling, behavioral studies, or pathogen surveillance [5,14,15]. Despite these functional differences and the interchangeable use of snap and cage traps as sampling tools for both research and control contexts [16], these tools require rigorous comparative evaluation to identify their specific strengths and weaknesses across different urban substrates to ensure proper deployment and data accuracy.

Previous studies using paired deployment of live and snap traps to compare trap performance across different species of small mammals in outdoor or natural habitats have reported species-specific trap biases, with snap traps capturing more larger-bodied species. However, within the same species, no weight bias was observed between the trap types [16]. Despite these findings, a comparative performance of these trap types for *Rattus norvegicus* in indoor urban environments remains poorly understood. In addition, prior research utilizing live-capture methods suggests that cage trap efficacy is not uniform across a population, but is instead influenced by individual characteristics such as sex, age, species and social status [16,17,18,19]. Because cage traps act as a selective filter, they may inadvertently bias data on population structure and pathogen prevalence [18,19,20]. However, it remains unclear whether lethal snap traps exhibit the same demographic biases or provide a more representative sample of the urban brown rat population.

The environmental context further complicates the trap performance. Substrate characteristics, such as permeable soils versus impermeable concrete, can influence rat movement, burrowing behavior, and encounter rates with stationary traps [2,21]. Structural placement also plays a key role; rats preferentially move along walls, burrow edges, and vertical infrastructure that function as locomotor pathways or runways, consistent with well-documented behaviors such as thigmotaxis [2,11]. Despite recognition of these behavioral tendencies, relatively few comparative trapping studies explicitly isolate trap-specific effects across different urban substrates (permeable soil and impermeable concrete surfaces). We posit that substrate type imposes distinct locomotor constraints: impermeable concrete forces animals to commute across exposed surfaces to access resources, whereas permeable soil allows for subsurface navigation and opportunistic emergence [2,22].

Comparative evaluations of commercially available rodent trap designs further underline the need for standardized field comparisons. Large-scale studies have demonstrated substantial variability in performance among snap trap designs, with Victor snap traps ranking among the most effective lethal options for larger rodent species [23]. As such, Victor snap traps are frequently used as a benchmark in applied rodent research. In contrast, single-capture cage traps remain widely used for live capture in urban environments, yet direct comparisons of cage and snap traps under contrasting indoor residential conditions remain limited. At the same time, current public health and rodent management programs continue to rely on long-standing trapping approaches, underscoring the need for improved evaluation and refinement of surveillance and control methods [1].

Here, we employed a paired-trap design to evaluate the performance of snap and cage traps across two distinct urban substrates, dirt-covered crawlspaces and concrete basements, within New York City, USA. Specifically, we aimed to: (i) examine whether trap type acts as a demographic filter with respect to sex and body mass; (ii) evaluate variation in capture success across structural placement areas; and (iii) compare the trap success rates. By isolating environmental and structural drivers of trap performance, this study provides empirically grounded guidance for urban rat surveillance, ecological inference, and urban rat management.

## 2. Materials and Methods

### 2.1. Study Sites

**Figure 1:**
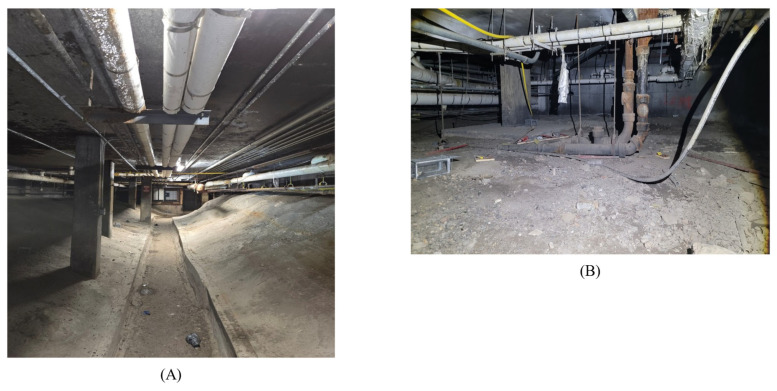
Apartment building basements and crawlspaces selected for the study. (**A**) A concreate-covered basement in Brooklyn. (**B**) A dirt-covered crawlspace in Queens.

This study took place in the basements and crawlspaces of residential buildings located in two New York City communities: three buildings in Queens and two buildings in Brooklyn (Figure 1). Site selection was based on recent reports of rat infestation from pest-control staff servicing the buildings and visual inspection confirming active *R. norvegicus* activity, including burrow openings, rat droppings, urine staining, sebum marks, gnaw marks, and other visible signs of rat activity within building basements and crawlspaces. The crawlspaces in the Queens buildings were characterized primarily by dirt substrates with many burrows and had an average area of approximately 64 m^2^ per building. In this study, dirt crawlspaces refer to enclosed, low-clearance sublevel service spaces beneath the sampled high-rise residential buildings, with exposed soil floors that allow burrowing. In contrast, basements in the Brooklyn buildings consisted predominantly (90%) of concrete floors with relatively less visible burrows, and were substantially larger, with an average area of approximately 264 m^2^ per building. In this study, the Brooklyn concrete basements refer to larger, continuous enclosed sublevel service areas within the sampled high-rise residential buildings, including concrete low-clearance sections that were not physically separated from the main basement areas. These areas mainly consisted of concrete floors that limits burrowing. Each basement contained two compactor rooms, a boiler room, and an electric meter room. T-Rex snap traps (Bell Laboratories, Inc. Windsor, WI, USA), and Catchmaster giant rat glue boards (AP&G Co., Inc., Bayonne, NJ, USA) were present in the Brooklyn buildings for rat control. In Queens, the CatchMor Ratinator Rat cage traps (63.5 cm × 38.1 cm × 16.5 cm) (Tractor Supply Co., Brentwood, TN, USA) and homemade bait containing Liqua-Tox II (Madison, WI, USA) were present in the crawlspaces for rat control.

Prior to the commencement of the trapping sessions, all pre-existing pest control devices within the study areas, including traps and bait sources, had either been removed or decommissioned in coordination with the housing authority to avoid interference with the study. These devices were inactive during the experimental trapping period, and no background trap captures occurred outside the experimental traps. At Queens, only one cage trap was previously present in one of the crawlspaces and was also decommissioned before the study period.

### 2.2. Trap Types

Two commonly used single-capture trap types were evaluated. The Victor^®^ rat snap trap (Woodstream Corp., Lititz, PA, USA) was selected based on prior evidence demonstrating high capture efficiency and suitability for *Rattus* spp. [23]. Cage trapping was performed using wire mesh live traps (Gingbau humane rat mouse cage trap 26.7 cm × 14.0 cm × 11.4 cm, available at amazon.com), which were used in previous rodent surveys [4,15]. Snap traps are lethal mechanical traps that use a spring-loaded bar triggered when a rat contacts the baited trigger plate. In contrast, live cage traps require the rat to enter an enclosed wire cage, where contact with the internal trigger mechanism closes the door and retains the animal alive until inspection.

### 2.3. Trap Deployment

**Figure 2:**
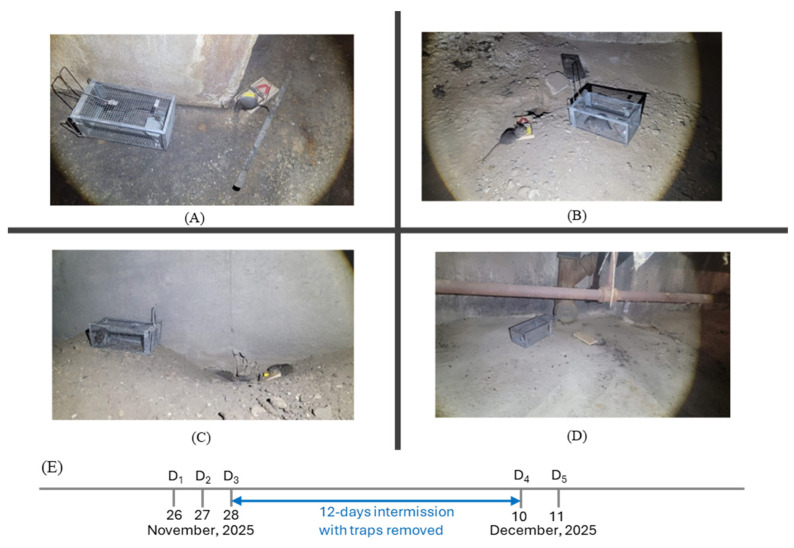
Trap placement areas and time line. (**A**) Floor, (**B**) burrow entrance, (**C**) wall-burrow, (**D**) vertical object, and (**E**) time line of trap deployment. All traps were triggered except the cage in (**D**).

Field research was conducted in two trapping sessions, defined here as discrete blocks of consecutive trapping nights within the same study period, totaling five nights between 26 November and 11 December 2025. The first session comprised three consecutive nights (26–28 November), followed by a 12-day intermission, during which the traps were unset and removed to allow for behavioral reset, with a second session of two nights (10–11 December 2025). On each sampling day, a total of 100 traps (52 in Queens, 48 in Brooklyn) were deployed, consisting of 50 snap traps and 50 cage traps, for a total of 500 trap nights (100 traps/night × 5 days) across five collection days. Traps were deployed in pairs, with each pair consisting of one snap trap and one cage trap placed approximately 30–60 cm apart (Figure 2). This paired-trap design ensured that both trap types were exposed to identical local conditions, including rat density, movement pathways, and environmental features. The trap locations within each pair were not switched between different days.

Pairs of traps were deployed in locations exhibiting signs of rat activity. These included active burrow entrances, at the perimeter of interior walls, between walls and burrows, at the base of vertical structural elements (e.g., pipes, pillars, and conduit runs), and floor areas with visible rat droppings, urine staining, or sebum marks. Placement locations were categorized into different types. The classifications differed slightly between Queens and Brooklyn sites to reflect differences in substrate and building structure. At Queens, which consisted primarily of crawlspaces with dirt substrates, the placement area categories included: (1) Wall-burrow: burrow entrances positioned along perimeter of walls; (2) Burrow: burrow entrances located in open floor spaces; (3) Vertical structure: vertical structural elements such as pipes, pillars, and conduit runs; and (4) Floor: open floor areas and wall edges with visible rat signs. At Brooklyn, which consisted of basements with concrete floor substrates, the placement area categories included: (1) Vertical Structure; (2) Floor; (3) Burrow (4) Compactor—the trash compactor room (2 rooms per basement). The number of traps placed in each area type varied by area size, microhabitat present or the presence of rodent signs. To reduce interference among trap stations and remain consistent with practical rat-control spacing recommendations, a minimum distance of approximately 3 m was maintained between trap pairs [24].

The snap and cage traps were baited with NoTox^TM^ soft bait attractant (Liphatech, Inc., Milwaukee, WI, USA). In addition, the cage traps also contained fresh apple slices in accordance with IACUC Protocol No. PROTO202400033. Traps were set during the day and were inspected the following day. At each check, the trap conditions were recorded as follows: not triggered, triggered without rat and triggered with a rat caught. Captured rats were removed from traps upon inspection. Rats captured in live cage traps were euthanized using CO_2_ inhalation via a 19-L plastic box as the fumigation chamber at the collection site. The snap traps and live cages were reset after each check.

Each trapped rat was weighed, sexed, and assigned to one of three age categories based on body mass: juvenile (<80 g), subadult (80–180 g for females; 80–200 g for males), or adult (>180 g for females; >200 g for males) [18,20,25].

### 2.4. Statistical Analyses

Capture success was modeled as a binary response variable (Captured = 1, not Captured = 0). Traps that were triggered without rat capture were treated as non-captures and were also coded 0. Differences in overall capture success between trap types were evaluated using Pearson’s Chi-squared test. Differences in capture success among placement areas were evaluated using Fisher’s exact test and adjusted using the Bonferroni correction. Generalized linear model (GLM) with binomial distribution was used to assess differences in sex ratios across locations and trap types. Temporal variations in trapping efficacy were evaluated using Fisher’s exact test. As body mass data were non-normally distributed, differences in body mass between trap types were assessed using the Mann–Whitney U test. All statistical analyses were conducted using R (version 4.5.1, R Foundation for Statistical Computing, Vienna, Austria).

## 3. Results

### 3.1. Overall Trapping Success

A total of 125 rats were captured during the five-day trapping period, yielding an overall capture success of 25% (125 rats divided by 500 trap nights). Only one rat was captured each time by either snap traps or by cages when they were triggered. There were three cage-trap events in which traps were triggered without rat capture; bait remained present in all three traps upon inspection, and these events represented 1% of the 250 cage trap nights. The capture success differed markedly between the two study sites. In Brooklyn, the overall capture success was substantially higher (38%) than that in Queens (13%) (χ^2^ = 42.4, df = 1, *p* < 0.001). This pattern was consistent across all buildings and was observed for both trap types. Within each site, there was no significant difference in capture success between snap and cage traps in Brooklyn (snap traps—40%, cages—37%) (χ^2^ = 0.28, df = 1, *p* = 0.595) and in Queens (snap traps—12%, cages—13%) (χ^2^ = 0.04, df = 1, *p* = 0.852) (Table 1).

Across 250 paired deployments for each trap type, both traps captured rats in 32 instances (13%). Cage traps captured rats alone in 29 instances (12%), whereas snap traps succeeded alone in 32 instances (13%). The trap success between the two types of traps was not statistically significant (χ^2^ = 0.07, df = 1, *p* = 0.80).

### 3.2. Capture Success at Different Placement Areas

In Brooklyn basements, the capture success varied significantly among the placement areas (χ^2^ = 23.04, df = 3, *p* < 0.001) (Table 2). The compactor room had significantly lower capture success rate (18%) than other areas (46–57%). In Queens crawlspaces, there were no significant differences in capture success among placement areas (χ^2^ = 6.50, df = 3, *p* = 0.090) (Table 2). Capture success at wall-burrows, burrow entrances, vertical structural elements, and floors is 19%, 13%, 11%, and 3%, respectively.

### 3.3. Demographic Bias Associated with Trap Type

A generalized linear model (GLM) with a binomial distribution showed a significant effect of trap type on the sex ratio (Z = −3.99, *p* < 0.001), with cage traps capturing a significantly higher proportion of females (90%) compared to snap traps (53%). There was no significant difference in sex ratio between the two study sites (Z = −0.31, *p* = 0.75), nor was there a significant interaction between site and trap type (Z = 1.05, *p* = 0.29), indicating that the female bias in cage traps was consistent across both environments.

Body mass distributions of the captured rats differed significantly between trap types at both sites (Table 3). Because body mass is not normally distributed, values are reported as the median with interquartile range (IQR). The median (IQR) body mass of rats caught by snap traps and cage traps in Brooklyn was 68 (57–93) and 56 (35–76) g, respectively. They are significantly different (U = 719.0, *p* = 0.009). Also, in Queens, the median (IQR) body mass of rats caught by snap traps and cage traps was 73 (55–208) and 49 (43–56), respectively. They are significantly different (U = 55.5, *p* = 0.004).

In addition, the age group demographics revealed a significant difference in the age composition of rats captured by each trap type. Snap traps captured a significantly higher proportion of mature individuals (subadults and adults) at 37.5% (24/64), compared to cage traps which captured only 20% (12/61) mature individuals (χ^2^ = 4.84, df = 1, *p* = 0.028). Consequently, cage traps captured significantly more juveniles (80%) than snap traps (62.5%).

### 3.4. Temporal Variation in Trapping Success

Temporal variation in capture success was assessed across the deployment days within the two trapping sessions of the study (Table 4). In the Brooklyn basements (concrete), there was an observed significant temporal variation in capture success. During Session 1, capture success decreased significantly from a baseline of 44% on Day 1 to 15% by Day 3 (Fisher’s exact test, *p* < 0.05). Following the 12-day intermission, capture success on the first day of Session 2 rebounded to 63%, which was significantly higher than the success rate at the end of the previous session (Fisher’s exact test, *p* < 0.01). This post-intermission success rate is statistically similar to that on Day 1 of the initial session (Fisher’s exact test, *p* = 0.101).

In the Queens crawlspaces (dirt), capture success followed a numerically similar pattern, declining from 19% on Day 1 to 6% on Day 3, and exhibiting a modest rebound to 13% after the intermission. However, due to the low overall capture volume, these temporal variations were not statistically significant across any of the deployment days (Fisher’s exact tests: *p* = 0.072 for the Day 1 to Day 3 decline, and *p* = 0.319 for the Day 3 to first day of session 2).

## 4. Discussion

### 4.1. Environmental Drivers of Trap Success

Our findings suggest that trap efficacy is modulated by local rat activity and environmental context. Basements in Brooklyn buildings yielded a nearly threefold higher capture success rate (38%) compared with crawlspaces in Queens (13%). The Brooklyn basements were larger than the Queens crawlspaces and had lower trap density than Queens, where capture success was lower. This suggests that the higher capture success in Brooklyn was likely a combination of greater local rat activity, substrate-related movement constraints, and trap density. The larger Brooklyn basements may have generally supported more rat activities [26,27], while the impermeable concrete substrate likely forced movement onto surface-based routes and structural bottlenecks where traps were placed. On concrete, rats are forced to traverse surface areas to access resources, increasing their encounter rates with stationary traps [21,28,29]. In contrast, the permeable dirt substrate at Queens allows for extensive subsurface burrowing, enabling rats to bypass surface traps [11].

Also, our result suggests that floor type could also dictate the spatial structure of rat movement. In basements with concrete floors (Brooklyn), we observed higher success rates when traps were placed around vertical objects (57%), on the floor (48%), and beside burrows (46%) compared to those in the compactor rooms (18%), even though rats were often seen feeding in compactor rooms. We attribute this to more risk aversion due to other available food resources from the compactor chute [18]. Also, the capture success in open placement areas could be attributed to the constraint of impermeability. On concrete, rats cannot create opportunistic exits; they are restricted to specific runways, structural defects or soil-access points (burrows), creating high-traffic bottlenecks that explain the higher success rate at these points. In addition, the relatively high success rate at vertical structures suggests that rats preferentially utilize pipes and pillars as vertical runways to exploit three-dimensional space and minimize surface exposure [2,11].

In the dirt-covered crawlspace environment (Queens), the ranking of capture success at different placement areas reveals a fundamentally different behavioral strategy. Wall-burrows were the highest-ranked area (19%), while crawlspace floors were the lowest-ranked (3%). The permeable soil allows rats to construct a dense network of burrow openings throughout the crawlspace, enabling them to surface close to their destination rather than commuting long distances [26,30]. Consequently, rats at Queens rarely traverse exposed crawlspace floors, likely explaining the near-zero capture success in these open placement areas. Instead, activity is spatially concentrated at the wall-burrow interface. This pattern is consistent with well-documented thigmotactic behavior [21] as rats prioritize movement along vertical edges. Furthermore, the use of burrow openings and vertical infrastructure allows rats to minimize predation risk [26] while securing safe portals to exterior food sources and adjacent structural voids [11].

These findings indicate that runway concepts should be adapted to the substrate. On concrete floors, the runway is essential, bridging distant entry or exit points, including cluster areas with heavy rat signs as a part of their foraging activities. On dirt floors, the runway is nearly non-existent on the open floor; instead, the critical interception points are the vertical structures and burrows, mainly those at the perimeter wall itself. Pest management strategies may therefore consider shifting from generic grid placements to targeted interception [31]: focusing on vertical infrastructure and burrow openings and runways in concrete basements, while prioritizing wall-burrow interfaces in dirt crawlspaces.

Nevertheless, the study was conducted from late November to mid-December, and the results should be interpreted within this seasonal context. In the northeastern United States, colder conditions may increase rat use of indoor refugia, such as basements and crawlspaces, for thermoregulation [11,27]. Furthermore, reduced outdoor food availability during winter months may force rats to increase their foraging risk, thereby increasing their interactions with baited traps [14,32]. Consequently, the capture success rates and demographic patterns observed in this study may partly reflect winter-associated conditions and should not be interpreted as a year-round baseline. Future studies conducted across multiple seasons are needed to determine how trap efficacy and demographic capture patterns vary with seasonal environmental conditions.

### 4.2. Trap Type as Demographic Filter

A critical finding of this study is that trap type acts as a significant demographic filter, challenging the assumption that cage and snap traps provide equivalent samples of local populations. Cage traps were heavily biased toward females (90% of pooled captures) and juvenile cohorts (median body mass 49–56 g). This supports a status-dependent risk aversion, in which larger or more dominant individuals control the territory and have access to familiar resources, while smaller or subordinate individuals may be more likely to interact with novel or higher-risk food sources [18,19,33]. Conventional live traps may also introduce a conspicuous structure into the environment and elicit neophobic responses, especially among older or more experienced individuals [18,33]. Although prior exposure to live-capture traps was limited in this study, information on the exact duration of pre-existing traps and glue boards at the study sites was unavailable. Therefore, historical experience with pest-control devices cannot be fully excluded as a behavioral influence. In contrast, snap traps captured a balanced sex ratio and significantly larger rats (median body mass 68–73 g). By utilizing a trigger-ready open architecture, snap traps placed along vertical structural runways (e.g., pipes and pillars) or emergence points (e.g., burrows, wall-burrows) exploit forced movement pathways [11,21]. In these contexts, heavier or more dominant males, despite their cage aversion, may not be able to avoid interceptor traps on their primary commuting route. Consequently, snap traps effectively bypass the behavioral filter by exploiting the physical constraints of the rats’ movement ecology.

Such bias has important implications for ecological inference and public health surveillance, as large adult rats, particularly dominant individuals, are more likely to contribute disproportionately to reproduction, movement across urban infrastructure, and the maintenance and transmission of zoonotic pathogens [20,25]. From a management perspective, reliance on a trap type that primarily captures younger or lighter-bodied rats may undermine control efficacy if mature reproductive individuals remain underrepresented [18]. Removing juvenile cohorts alone may have limited long-term impact on population suppression if older, established individuals continue to evade capture and contribute disproportionately to reproduction and population persistence [25,26].

Nonetheless, established adults exceeding 350 g were limited in our captured sample at 3.2% of total captures across both substrates. Under an urban-framework interpretation, these heavier individuals may represent more established or resident rats. Therefore, the applicability of these trap-associated demographic patterns to larger, established adults should be interpreted cautiously. Such individuals may exhibit stronger neophobia, greater behavioral restraint around novel devices, and greater access to familiar resources through social dominance, potentially reducing their susceptibility to both cage and snap traps.

### 4.3. Temporal Variation

Consistent with patterns observed in our other field trapping efforts, we observed a decline-reset-rebound pattern in daily capture success over the two trapping sessions (Table 4). This temporal dynamic was statistically significant in Brooklyn basements, where capture success decreased from 44% on Day 1 to 15% by Day 3, then rebounded to 63% following a 12-day cessation period during which traps were removed. In Queens crawlspaces, we also observed a decline during the first session followed by an increase in the second session; however, this pattern was not statistically significant, likely due in part to the lower overall capture volume.

Temporal rebounds in capture success may reflect the reversal of short-term behavioral avoidance of trapping devices [9,11], compensatory immigration, and increased local activity of previously uncaptured rats, all of which are plausible contributing factors. Given our trap density, intensive trapping may have permitted a rapid local population depletion in the first session [34], creating vacant space or reduced competition that allows previously uncaptured residents or rats from adjacent areas to enter or increase activity within the sampled area [14,18].

To further explore this pattern, we compared body mass between trapping sessions. In Brooklyn, where the temporal rebound was significant, median body mass declined significantly from 74.35 g in Session 1 to 57.85 g in Session 2 (Mann–Whitney U test, *p* = 0.002). This shift suggests that the second trapping session captured lighter-bodied individuals than the first session, which is consistent with initial depletion of the heavier or socially dominant individuals, allowing the remaining smaller, subordinate individuals to be trapped while more freely exploring for resource acquisition [14,18,26]. However, without individual identification, we cannot determine whether these individuals were immigrants, previously uncaptured residents, or newly active individuals [3].

In Queens, median body mass did not differ significantly between sessions, declining only slightly from 59.0 g to 54.2 g (Mann–Whitney U test, *p* = 0.507). This lack of a significant demographic shift suggests that the crawlspace environment may have produced different temporal trapping dynamics than the concrete basements. The permeable dirt substrate and extensive burrow systems may have allowed rats to reduce encounters with surface traps, limiting both depletion of the trappable population and any detectable post-intermission shift in capture demographics.

From an applied perspective, this finding suggests that short, pulsed trapping deployments may be more effective than continuous trapping, a hypothesis that warrants formal testing in future studies [11].

## 5. Conclusions

In concrete environments, the number of burrow openings is very limited, and rats are forced to commute across open floors and vertical infrastructure, which makes them vulnerable to interception at these bottlenecks. Conversely, in dirt-covered crawlspaces, numerous burrows are present, creating many wall-burrow interfaces, making open floor areas less productive for rat trap placement. Capture success increased after the 12-day cessation period, but this rebound may reflect multiple non-mutually exclusive mechanisms, including behavioral avoidance, increased local activity of previously uncaptured rats, or compensatory immigration. Furthermore, cage traps produced a more female-biased and lighter-bodied sample, whereas snap traps captured a more balanced sex ratio and relatively heavier individuals. This trap-associated demographic bias is important because reliance on a single trap type may provide a skewed view of population structure and potential pathogen risks. Therefore, we propose the future rat trapping tactics: (1) tailor trap placement to the building environment; (2) employ a mixed-method approach, such as using both snap traps and cage traps, to maximize capture success across all demographic cohorts. However, these findings should be interpreted within the context of high-rise residential buildings in New York City and may not directly generalize to other cities, building types, climates, or urban management settings.

## Figures and Tables

**Table 1:** Overall trapping efficacy by site and trap type.

Site	Trap Type	Number of Trap Nights	Number of Captures *	Success Rate	Pearson’s χ^2^ Test
Brooklyn	Snap	120	48	40%	χ^2^ = 0.28, df = 1, *p* = 0.595
	Cage	120	44	37%	
Queens	Snap	130	16	12%	χ^2^ = 0.04, df = 1, *p* = 0.852
	Cage	130	17	13%	

* Only one rat was captured each time by either snap traps or by cages when they were triggered.

**Table 2:** Variations in capture success among placement areas.

Site	Placement Area	Number of Trap Nights	Total Success (%) *	Cage Success (%)	Snap Success (%)	Fisher’s Exact Test (*p*)
Brooklyn	Vertical Object	30	57% a	53%	60%	1.000
	Floor	80	48% a	48%	48%	1.000
	Burrow entrance	50	46% a	40%	52%	0.571
	Compactor room	80	18% b	18%	18%	1.000
Queens	Wall-burrow	80	19% a	10%	28%	0.083
	Burrow entrance	70	13% a	17%	9%	0.477
	Vertical object	70	11% a	17%	6%	0.259
	Floor	40	3% a	5%	0%	1.000

* Within each site, numbers followed by the same letters are not significantly different (Fisher’s exact test with Bonferroni correction, *p* > 0.05).

**Table 3:** Body mass (g) of captured rats.

Site	Trap Type	Median (IQR) Body Mass (g) *	Statistics
Brooklyn	Snap	68 (57–93)	U = 719.0, *p* = 0.009
	Cage	56 (35–76)	
Queens	Snap	73 (55–208)	U = 55.5, *p* = 0.004
	Cage	49 (43–56)	

* IQR (Interquartile Range) represents the middle 50%.

**Table 4:** Temporal variation in trapping success across two consecutive deployment sessions.

Trapping Session	Deployment Day	Brooklyn Trap Nights	Capture Success (Brooklyn) *	Queens Trap Nights	Capture Success (Queens)
Session 1 (26–28 November 2025)	Day 1	48	44% a,b	52	19%
	Day 2	48	25% b,c	52	13%
	Day 3	48	15% c	52	6%
Intermission	12-Day break	–	–	–	–
Session 2 (10–11 December 2025)	Day 1	48	63% a	52	13%
	Day 2	48	46% a,b	52	12%

* Numbers followed by the same letters are not significantly different (Fisher’s exact test, *p* > 0.05).

## Data Availability

All research data in this study are available on request from the corresponding author.
